# Distinct role of autophagy on angiogenesis: highlights on the effect of autophagy in endothelial lineage and progenitor cells

**DOI:** 10.1186/s13287-018-1060-5

**Published:** 2018-11-08

**Authors:** Mehdi Hassanpour, Aysa Rezabakhsh, Masoud Pezeshkian, Reza Rahbarghazi, Mohammad Nouri

**Affiliations:** 1 Department of Clinical Biochemistry and Laboratory Medicine, Tabriz University of Medical Sciences, Tabriz, Iran; 2 Stem Cell Research Center, Tabriz University of Medical Sciences, Imam Reza St., Golgasht St., Tabriz, 5166614756 Iran; 3 Stem Cell And Regenerative Medicine Institute, Tabriz University of Medical Sciences, Tabriz, Iran; 4 Emergency Medicine Research Team, Tabriz University of Medical Sciences, Tabriz, Iran; 5 Department of Applied Drug Research, Tabriz University of Medical Sciences, Tabriz, Iran; 6 Department of Applied Cell Sciences, Faculty of Advanced Medical Sciences, Tabriz University of Medical Sciences, Tabriz, Iran

**Keywords:** Stem cell, Autophagy, Angiogenesis, Differentiation, Functional maturation

## Abstract

Autophagy plays a critical role in the dynamic growth of each cell through different conditions. It seems that this intracellular mechanism acts as a two-edged sword against the numerous cell insults. Previously, autophagy was described in the context of cell activity and behavior, but little knowledge exists related to the role of autophagy in endothelial cells, progenitors, and stem cells biology from different tissues. Angiogenic behavior of endothelial lineage and various stem cells are touted as an inevitable feature in the restoration of different damaged tissues and organs. This capacity was found to be dictated by autophagy signaling pathway. This review article highlights the fundamental role of cell autophagic response in endothelial cells function, stem cells dynamic, and differentiation rate. It seems that elucidation of the mechanisms related to pro- and/or anti-angiogenic potential of autophagy inside endothelial cells and stem cells could help us to modulate stem cell therapeutic feature post-transplantation.

## Terms and definition

**Fig. 1:**
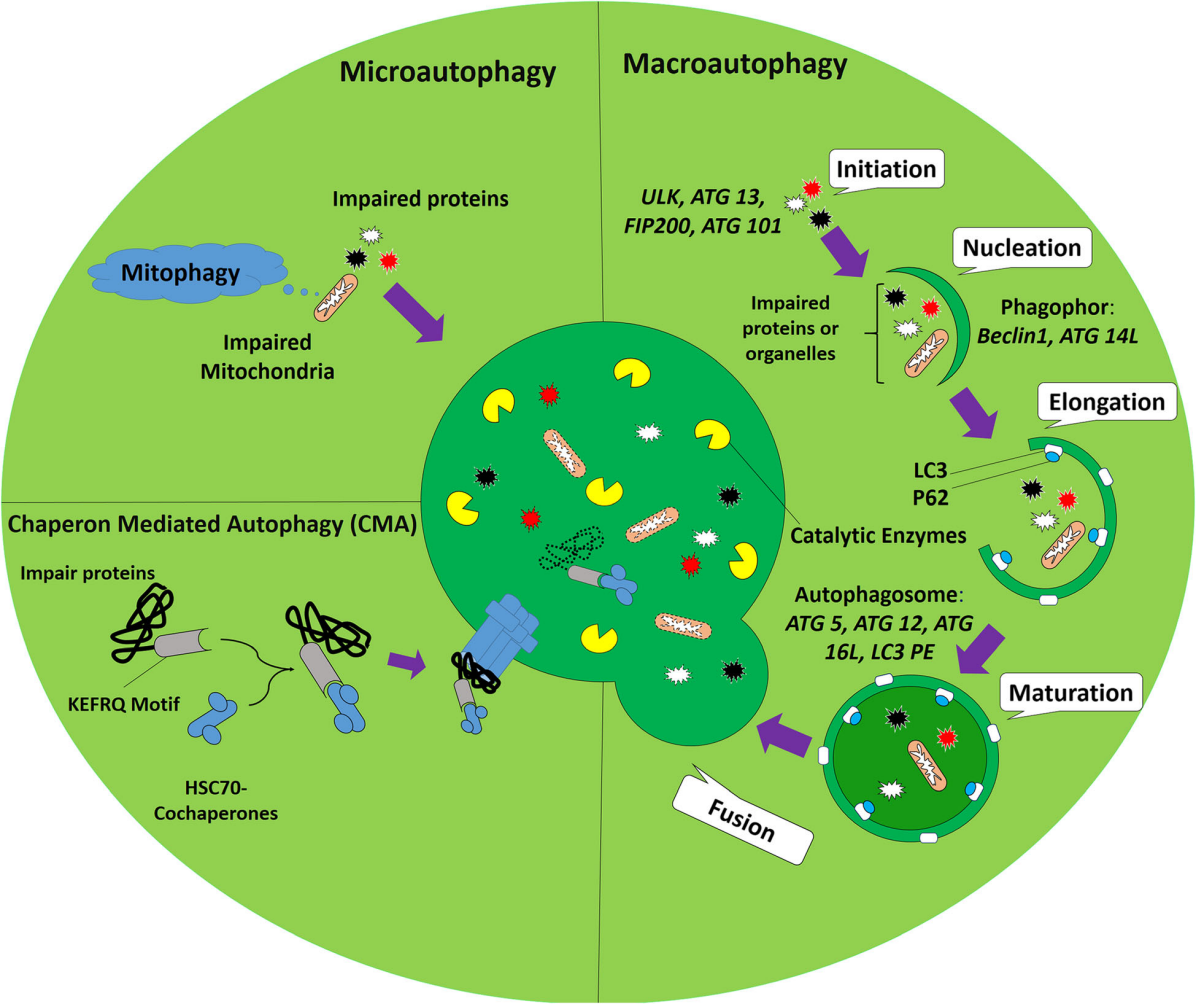
Autophagy is classified into three types based on the route of delivery. Microautophagy refers to the sequestration of misfunction proteins or whole organelles such as mitochondria (named mitophagy) directly by lysosomes. Chaperone-mediated autophagy (CMA) involves direct translocation of misfolded substrates across the lysosome membrane through the action of a cytosolic and lysosomal chaperone hsc70, and the integral membrane receptor LAMP-2A (lysosome-associated membrane protein type 2A). In the case of macroautophagy, the cargoes are sequestered within a unique double-membrane cytosolic vesicle, named autophagosome. This type of autophagy is initiated by the nucleation of an isolation membrane or phagophore. ULK, ATG 13, FIP200, and ATG101 are involved in this stage. Then, the Beclin-1 and ATG14L complex contributes to the nucleation of the phagophore. This membrane then elongates and closes on itself to form an autophagosome. Elongation of the phagophore membrane is dependent on the Atg12 and LC3 conjugation systems. Closure of the autophagosome is dependent on the activity of the LC3-conjugation system. The autophagosome matures by fusing with endosomes and lysosomes, finally forming the autophagolysosome where the cargo degradation occurs

In addition to synthesis, protein degradation is an important mechanism for the physiologic activity of each cell inside the body. Cells commonly exploit two main strategies to degrade intracellular proteins, namely a ubiquitin-proteasome system and autophagy [1]. Autophagy is apparently a crucial and tight regulated normal catabolic process for cell survival during growth, starvation, and differentiation and even plays fundamental biological roles in various cellular functions [1]. In scientific literature, autophagy is defined as sequestration of misfolded, impaired, and toxic aggregate-prone mutant proteins, whole damaged and dysfunctional organelles, or intracellular pathogens into double-membrane autophagic vesicles termed autophagosomes that finally merge with lysosome for degradation (autophagolysosome) [2]. Regarding delivery pathway of the biomolecules to the lysosomes, three distinct types of autophagy mechanisms were previously discovered: (a) Macroautophagy that surrounds the material with a dual lipid membrane, named autophagosome and then fuses with lysosomes finally formed autophagolysosome; (b) microautophagy, which directly enters substances into the lysosomes through the intrusion of self-membrane (Fig. 1); and (c) CMA that is degradation of a proteins with specific motifs (KFERQ) targeted by HSC70 complex and then adhere to lysosome via LAMP2A (Fig. 1). Other aliases exist regarding autophagy such as aggrephagy, mitophagy, lipophagy, and xenophagy depending on which substance is sequestrated and digested [3, 4]. In this review, macroautophagy will be termed as autophagy. Autophagy is a general response to a diversity of external and internal stress stimuli. Under these conditions, autophagy is stimulated via signaling the mechanisms that ordinarily involve activation of the AMPK and inhibition of mTOR [5]. The process of autophagolysosome formation involves initiation, elongation, and maturation stages and the following merge of the autophagosome with lysosomes to form an autophagolysosome [2]. Autophagy is initiated by a membrane nucleation that requires the ULK1 complex with FIP200, Atg13, Atg9, a regulatory class III PI3K complex that includes Beclin-1, known as Atg6, and Atg5-Atg12-Atg16 multimerization complex [6] (Fig. 1). Under physiological conditions, mTOR prevents autophagy by the phosphorylation and inhibition of Atg13 activity that inhibits its interaction with ULK1. mTOR also prevents ULK1 activity by phosphorylation of ULK1 and disrupting the interaction between ULK1 and AMPK [7]. In the absence of these inhibitory signals, Atg13 and ULK1 interact with Atg17 and the ULK1-Atg13-Atg17 complex to initiate autophagosome formation. Recruitment of Atg9 to the ULK1-Atg13 complex is a key step for the primary lipidation of pre-autophagosomal structure membrane [8]. The next steps are regulated by a complex of Beclin-1 with PI3K-III and Atg14 [9]. As soon as autophagy is stimulated by starvation and hypoxia, Atg14 (the pre-autophagosome protein) accumulates at the endoplasmic reticulum-mitochondrion contact surface in a SNARE, syntaxin 17-dependent manner [10]. Atg14 connects PI3K molecules to Beclin-1 and results in the development of a complex on the pre-autophagosomal structure [10]. Afterward, many of the Atg proteins will focus on the pre-autophagosomal structure; therefore, the pre-autophagosomal structure is considered to be linked to the formation of the autophagosomes [11]. Moreover, Beclin-1 is released from Bcl-2 and forms a complex with the ultraviolet radiation resistant gene/activating molecule in Beclin 1-regulated autophagy [12]. This complex starts conjugation of Atg12 to the substrate Atg5 by Atg7 and Atg10 to form an Atg5-Atg12-Atg16 multimeric complex [13]. Atg4 converts LC3 from LC3β-I, free-form, to LC3β-II, in phosphatidylethanolamine-conjugated form, that regarded as a crucial step in autophagosome formation. The LC3β-II is conjugated via phosphatidylethanolamine irregularly on both sides of the membrane by Atg9 within the ULK complex [14]. This process will more continue until the end of the autophagosome formation. So, LC3β-II is released from the external surface of the membrane. Then, from this conception, it can be concluded that LC3β-II can serve as an analytical marker for monitoring autophagic flux [15]. The newly formed autophagosome together with the cargo to be degraded finally merge with lysosomes to form an autolysosome that their content is lysed by lysosomal enzymes [2]. By transferring the autophagosomes to lysosomal proximity, microtubules regulate and facilitate the fusion of autophagosomes with lysosomes. Then, LAMP1/2, lysosomal membrane proteins, Rab7 (member of Rab GTPases family), SNARE, class III Vps, and endosomal sorting complexes are required for transport which mediates the process of fusion [16, 17]. Finally, the formation of the autolysosome provides an acidic environment that is essential for the ideal activity of lysosomal hydrolases, cathepsins and cargo degradation [18]. Amid autophagy factors, p62 is degraded along with the targeted proteins and organelles, while LC3 may be degraded or recycled back into the cytosol pool [19]. It is noteworthy to conclude that the pattern of autophagy proteins reveals the state and progression of autophagy. Activation of autophagy contributes to the accumulation of LC3-bound, increased LC3-II/I ratio, and reduction of p62 levels, reflecting degradation in the autolysosomes [20]. The defect of autophagy at the beginning stage is characterized by a decreased LC-3-bound, loss of LC3-II, and raised p62 levels inside cells [21–23]. Failure of the final phases of autophagy, autophagosome-lysosome fusion or cargo degradation, is characterized by a normal or increased number of LC-3-bound, increased LC3-II and p62 in the cell that revealed a failure to clear autophagosomes and degrade p62 (Fig. 1) [22].

## Effect of autophagy on various SC differentiation

Despite distinct features of self-renewal, differentiation, multi-potency, and quiescence status in different organs, SCs should improve the restorative potency of organelles turnover [24]. In this regard, autophagy as a well-known quality control process is essential for the high maintenance of SCs homeostasis [25]. Based on their origin, SCs could be classified into two types and one of them is ESCs that is originated from inner cell mass of murine and human blastocytes and the second one is ASCs seen isolated from adult tissues. Compare to ESCs, ASCs, but not all types such as intestine crypt stem cells, pose a limited stemness feature which is localized in specific mature sites to repair tissues in response to certain diseases [26, 27]. Maintenance of balance between stemness and differentiation in SCs is a very important issue [28]. An excessive differentiation rate may lead to cell aging, while untamed cell proliferation can raise the emergence of cancer cells formation [29]. Growing body of evidence revealed that autophagy modulates differentiation in both ESCs and ASCs [30]. It seems that autophagy is one of the indirect metabolic pathways that can be modulated in both ESCs and ASCs. Thus, we briefly scoped the crucial role of autophagy response in the maintenance of stemness and differentiation of various SC types. Commensurate with these descriptions, autophagy response and related signaling pathways have potential to direct and inspire distinct phenotype and cell function in SCs in the favor of regeneration or reduce restorative capacity.

### Role of autophagy on ESCs

Autophagy has an essential role during cell reprogramming and embryogenesis period [31]. Autophagy response is commonly upregulated at an early stage of human and murine SC differentiation [32]. In line with this statement, some pharmacologic agents are used for deciphering autophagy effects on the differentiation capacity of SCs [33, 34]. HMBOX1, known as a transcription repressor, has the ability to regulate autophagy [35]. Inhibition of HMBOX1 could per se inhibit the autophagy while the promotion of this factor stimulates autophagy flux [35]. It has been reported that during MSC and ESC differentiation into endothelial-like cells, the stimulation of HMBOX1 coincided with the induction of autophagic response [35]. It has been elucidated that HMBOX1 potentially interacts with MT2A to initiate autophagy while inhibiting apoptosis in vascular ECs [35]. Under treatment with autophagy inducer such as rapamycin ESCs transition into osteoblast cells was promoted by the inhibition of mTOR and promotion of BMP/Smad signaling pathway [36]. Autophagy also increased human ESCs’ survival by SIRT-1 activation and subsequent mTOR inhibition against oxidative stress-induced damages [37]. The factor SIRT1 was found to stimulate autophagy-related signaling pathway and improve mitochondria function in ECs under oxidative stress. There is a close relation with autophagy and cytokines participating in stemness acquisition [38, 39]. For instance, it was reported that LIF is required to preserve the multi-potency of murine ESCs [38, 39]. In vitro depletion of LIF from medium has the potential to trigger mTOR signaling pathway through the modulation of ERK/Tuberin axis [40]. LIF depletion also contributes to the activation of MEK/ERK/TSC2 transition from the LIF/STAT3-induced pluripotent state to the fibroblast growth factor receptor 2/ERK-committed differentiation governed by mTOR signaling activation [39]. In an experiment, it was shown that *Atg3*^+/−^, *Atg3*^−/−^, and *FOXO-1*^−/−^ murine ESCs lost the potency of self-renewal and differentiation capacity [41]. The high autophagic flux rate ensures ESC entity through engaging autophagy machinery effector termed FOXO-1. This factor acts a pivotal role in the regulation of SOX2 and OCT4 (POU5F1) expression [41, 42]. There is an inevitable correlation between FOXO-1 level and the expression of autophagy-related genes. The deficiency of autophagy genes such as *Atg5* and *Beclin-1* has long been reported ESCs during embryogenesis and fetal development [43]. Both genes participate in SC cavitation during the formation of the embryoid body [43]. Even, modulating autophagy under insulting condition could have some beneficial outcomes regarding SC orientation toward specific phenotypes.

During starvation, the induction of autophagy via the treatment of rapamycin could induce morphological changes by degrading the midbody ring prior to cell-to-cell separation [44]. By using *Atg5*-null oocytes and sperms, the generation of the blastocyst and therefore inner cell mass was stopped [45]. Commensurate with these comments, the ablation of Beclin-1 gene leads to early embryonic lethality in murine ESCs [46]. Some compensatory mechanism could be activated in autophagy-deficient conditions. For example, the ubiquitin-proteasome system degrades the damaged organelles and unfolds proteins in response to autophagy insufficiency [47]. This reciprocal crosstalk between the ubiquitin-proteasome system and autophagy signaling has been documented in human autophagy-deficient ESCs. To further follow autophagic state on cell differentiation capacity during early embryogenesis, HESC-GFP-LC3 cell line was established based on the expression of the green fluorescent protein encoded with some pluripotency markers [48]. Ambra1 deficiency, as a positive regulator of autophagy, may cause embryonic lethality and lack of Ambra1 function, increased apoptotic changes and predisposed nervous system insufficiency [49]. Overall, autophagy is an essential element for an early embryogenesis and differentiation during the development of the embryo. By controlling the effectors through autophagy signaling pathway, we are able to modulate the differentiation and orientation of distinct SCs toward specific lineages.

### Role of autophagy on HSCs

There are a plethora of experiments implicating the role of autophagy on the dynamic hemostasis of HSCs. HSCs can differentiate to blood progenitor cells and restore all blood cell types. In the absence of autophagy, differentiation of HSCs into mature lineages was disrupted and the possibility of malignancies would increase [50]. For instance, in null Atg5^−/−^ mice, an increased cell apoptosis in cytotoxic CD8^+^ T cells was observed with the occurrence of lymphopenia [51]. Other authors demonstrated that mutation of another autophagy member Atg7 in hematopoietic system yielded an impaired mitophagy by enhancing of ROS generation, oxidative stress and DNA damage in erythrocytes with subsequent anemia and defective self-renewal [50]. The application of siRNAs targeting Atg 5 and Atg 7 fails to generate colony formation in HSCs in vitro [32]. These results show that autophagy mainly preserves self-renewal and multipotency of HSCs in human or murine models [32]. The factor LKB1, a Serine/threonine-protein kinase tumor suppressor, has an indispensable role in maintaining energy hemostasis [52]. Lack of LKB1 in mouse HSC leads to cell cytotoxicity and accumulation of LC3-II in the bone marrow, spleen, and thymus [53]. Recent studies confirmed that the modulation of miRNAs, miR-17, − 20, − 93, and − 106 have the potency to regulate the dynamic growth of HSCs through the downregulation of gene *p62* [54]. P62, an adaptor protein, plays a key role in proteasomal and autophagy degradation pathway [55]. Based on the data from recent studies, autophagy role was shown in the last stages of HSC differentiation [54]. Some factors could control the activity of autophagy-related genes [56]. For instance, GATA1 as a crucial regulator of the hematopoietic system could also control *MAP1LC3B* as well as lysosomal biogenesis factors [56]. Recent findings declared that transcription of autophagy-related genes was enhanced during fetal HSC differentiation in murine embryo indicated by single-cell RNA sequencing technique [57]. In Tie2^+^ HSCs, mitophagy was found to have an essential role in impaired mitochondria clearance and the maintenance of stemness feature of murine HSCs [58]. By inducing PINK1 and PRKN genes, two key regulators of mitophagy, the differentiation property of HSCs was confirmed in high levels [58]. Deletion of PINK1 and PRKN genes caused a failure in the regeneration and renewal activity of HSCs. Regarding these comments, it is noteworthy that autophagy is an essential element for stable quiescence in HSCs [59]. This finding strongly supports a notion that autophagy not only has a pivotal role in multipotency and remodeling of HSCs under physiologic condition but also preserves stemness of HSCs by decreasing oxidative stress [60]. Interestingly, autophagy activity is touted as an important mechanism to suppress HSCs metabolism and preserve stemness with aging [61]. The regulation of basal cell metabolism and function of young and old SCs is done via engaging autophagy-related effectors [61]. A factor titled FOXO3 activates a Bcl-2 interacting mediator of cell death and promotes mitochondrial depolarization and subsequent ROS generation. The activation of FOXO3 could prohibit ROS production by survivin activation and BCL-XL inhibition. Notably, FOXO3A regulates a pro-autophagy gene expression status to maintain HSCs by autophagic responses following the occurrence of metabolic stress [62]. Mutant Beclin-1^+/−^ or Atg5^−/−^ HSCs trigger the upregulation of Bcl-2 expression, causing genomic instability, aneuploidy, and DNA and chromosomal damages [63]. A protective effect of autophagy on HSC genomic integrity and reconstitution capacity was indicated in irradiated mice [64].

### Role of autophagy on NSCs

Evidence point NSCs could proliferate and differentiate into other types of neural lineages. For the first time, the effect of autophagy was investigated on in vitro model of murine neuroblastoma cell line N2a cells [65]. Similar to other stem cell types, the critical role of FOXO1, FOXO3, and FOXO4 have been documented on the dynamics of murine NSCs. For instance, in *FOXO1*, *FOXO3*, and *FOXO4* null mice, oxidative stress and uncontrolled ROS production abrogated NSCs’ proliferation and inhibited the NSC differentiation potential [66]. *Atg9a* high expression rate (synaptic proteins) induced by miR-34a downregulation was shown to affect the murine NSC differentiation feature [67]. Another study confirmed that deletion of ULK-interacting protein FIP200 required for autophagosomes increased ROS content and superoxide level during p62 aggregation. These features promoted NSCs cell death through p53-dependent apoptosis pathway and cell cycle arrest [68]. Under in vivo condition, FIP200 is also required for NSC differentiation in the sub-ventricular zone of neonatal mice by the simultaneous limitation of microglia activity [69]. Suppression of SIRT1, a member of the sirtuin family, also could impress the NSC differentiation as well [70]. The expression of MiR-34a reduced SIRT1 expression and enhanced NSCs maturation rate and differentiation potential [71]. These findings support the notion that autophagy has a crucial role in NSC differentiation. A large number of experiments proposed the role of autophagy on embryonic and adult neural stem cells. According to recent findings, the expression of *MapLC3a, Belcin-1, Atg7*, and *Ambra1* were significantly increased in in vitro model of murine olfactory bulb-derived NSCs [72]. In *Ambra1* knockout mice, neural differentiation was abrogated and followed by neural tube defects during embryogenesis [49]. By suppressing *Atg5* in the murine cerebrocortical region, the defective neurogenesis was observed [73]. Chemical induction of SC differentiation promoted the increased GFP-LC3 punctae and genetic/chemical inhibition of autophagy caused defective differentiation in N2a cells [65]. In the autophagy-related pathway, the role of lysosome in degradation is not indispensable. Then, lysosomal dysfunction leads to neural or non-neural death because of extra-accumulation of autophagosomes [74]. It has been posted in an experiment that the deletion of gene *Atg7* could also amplify the neural cell death caused by chemical inhibition of autophagy at a late stage [75]. Ataxia telangiectasia mutated protein kinase has a key role in response to DNA damages and impaired genomic integrity. In Ataxia telangiectasia-mutated protein kinase-deficient NSCs, oxidative stress and ROS accumulation appeared by p38-MAPK activation and p16 induction [76, 77]. Considering dual roles of autophagy on cell dynamic, recent findings proposed that the infection of human fetal NSCs by Zika virus via NS4a A and NS4B (two important Zika proteins) inhibits Akt-mTOR signaling and induces abrupt autophagy, leading to defective neurogenesis and cell death [78]. Consistent with such an idea, autophagy also serves as a quality control mechanism in the hippocampal region by preserving stemness and neurogenesis rate. Also, autophagy has the potential to eliminate defective NSCs following insulin withdrawal by type 3 ryanodine receptor-mediated endoplasmic reticulum Ca^2+^ regulation of autophagy and programmed cell death in NSCs [79, 80].

### Role of autophagy on MSCs

MSCs are pluripotent SCs that can differentiate into a variety of cell types including osteoblasts, chondrocytes, fibroblasts, adipocytes, and ECs. During differentiation to various cell lines, some factors are required for the osteogenic-adipogenic switch. For example, culturing density, cell shape, stimulatory growth factors, structural properties, and the levels of Rho GTPase (Rho-A) activity seems to be important [81, 82]. Although there is a little knowledge about autophagy role on MSCs, some studies demonstrated that autophagy has an essential role in oriented differentiation of MSCs and touted as a therapeutic value especially in regenerative medicine. The more recent experiment confirmed that autophagy induction sustains MSCs survival rate and promotes MSCs proliferation and differentiation into neural-like cells by increasing neuron-specific enolase and microtubule-associated protein 2 expression [83]. In pre-adipocytes, *Atg7* and *Atg5* mutations caused the reduction of adipose mass and differentiation, and an enhanced insulin sensitivity of *Beclin-1* promoted differentiated chondrocytes cell death [84]. Autophagy also could protect mesenchyme-derived chondrocytes during the occurrence of osteoarthritis through engaging *ULK1*, *BECN1,* and *MAP1LC3A* [85]. Likewise, constitutive autophagy response plays an essential role in osteocytes hemostasis. Silencing the RB1CC1/FIP200 complex genes, essential for autophagosome formation, caused disruption of osteogenesis in osteoblasts in vitro and in vivo models [86]. Moreover, autophagy participates in osteogenic differentiation of human dental pulp derived-MScs time-dependently through mTOR inhibition-mediated autophagy and late activation of Akt/mTOR signaling pathways [87]. It is important to note that competent autophagy activity is limited to undifferentiated MSCs. Recent data showed that the level of GFP-LC3 puncta in primary murine bone marrow stromal cells isolated from GFP-LC3 transgenic mice is significantly low after differentiation into osteoblast cells [86]. Moreover, the authors demonstrated that autophagy induction protected primary and cell line MSCs from apoptotic cell death under the hypoxic condition and serum deprivation by releasing anti-apoptotic or pro-survival factors [88, 89]. Contradictory findings revealed the modulatory effect of autophagy in MSC-based cell therapy. In better words, the suppression of autophagy could accelerate the liver regeneration in *the* in vivo model of acute liver failure [90]. Interestingly, the quality control of autophagy plays an indispensable role in neurodegenerative diseases such as Alzheimer and Parkinson. Vice versa, a recent finding demonstrated the interactive role of MSCs against autophagy machinery system. In fact, MSCs enhance autophagic activity and subsequently β-amyloid clearance in Alzheimer’s disease [91]. SIRT1 protein as one of the key modulator of autophagy machinery influences the MSC differentiation. SIRT1 can promote the mesangial cell proliferation under high glucose condition and restrict expression of aged MSCs phenotypes. Mutation of miR-195 in aged MSCs enhanced SIRT1 function and anti-age relating factors including FOXO1, Akt, and telomerase reverse transcriptase [38, 92, 93].

### Role of autophagy on MPCs and CPCs

The skeletal muscles are polynucleated syncytial cells which composed of myofibers and carry out the growth and the repair of muscle cells. The existence of MPCs known as satellite cells plays a crucial on muscle restoration [94]. During muscle development, embryonic myoblasts differentiate to adult myofibers and constitute the mature muscles. These myofibers are responsible for the further development of neonatal myogenesis [94]. Satellite cells are entirely quiescence while after muscle damage fused to damaged muscles and stimulate the muscle proliferation and differentiation. The effect of autophagy on MPCs just has been investigated recently. García-Prat and co-workers showed that the population of murine satellite cells, in Atg-7 (^−/−^) mutant cells notably reduced [95]. In agreement with this finding, in aged satellite cells, a similar phenotype of Atg7-deficient cells was observed coincided with enhanced senescence markers such as p16INK4a, P15INK4b, p21CIP1, and increased oxidative stress. Additionally, DNA damage and p62 aggregation were imitated. Moreover, reduction of autophagic flux was detected in satellite cells isolated from aged mice [96, 97]. Autophagy regulates satellite cells’ bioenergetics feature during cell function and circadian recycling of cell damaged ingredients and organelles [98, 99]. CPCs have the ability to differentiate into ECs and smooth muscle cells besides cardiomyocytes. Suppression of FGF signaling axis is required for CPC differentiation capacity [100]. By activating FGF receptors, autophagy machinery was inhibited by the engaging Akt-MAPK pathway. On the other hand, the induction of autophagy, in turn, blocks FGF receptors and promotes the CPC differentiation. Due to the role of autophagy on maintenance of CPCs survival, autophagy induction is one of the most important strategies besides the SCs derived exosomes and expression of miRNAs in SC therapy of ischemic myocardium [101].

### Role of autophagy on CSCs

Recently, most of the studies focused on autophagy role in cancer stem cell characteristics [102, 103]. In a comparison of other stem/progenitor cells, CSCs have a similar self-renewal property but a higher level of basal autophagy activity especially under hypoxic niche beside of metastasis potency and resistance against chemotherapy [104, 105]. Considering the dual role of autophagy in either tumor-suppressing or tumor-promotion, autophagy effect on CSCs strongly correlates with cancer type and the stage of tumor development [106]. In the case of tumor suppression, the contradictory data implicated that mutation of the PTEN gene emerged during malignant differentiation of SCs while PTEN downregulation is associated with repopulation and tumorgenesis capacity of CSCs [107–109]. The high content of PTEN promotes autophagy induction and limits the CSC activity. In contrast, autophagy inhibition by the silencing of *BECN1* and *ATG7* genes was found to abrogate the self-renewal of breast cancer cells both in vivo and in vitro models [110, 111]. In an experiment, it was unveiled that FIP200, as a very important regulator of autophagy, reduced the tumor-propagating of both aldehyde dehydrogenase 1 positive and CD29^hi^CD61^+^ breast CSCs through suppressing the EGFR/Stat3 and TGF-β/Smad axis [83]. Moreover, loss of autophagy reduced survival rates of chronic myeloid leukemia CD34^+^ progenitor cells while promoting the acute myeloid leukemia progenitor cells proliferation [103]. Yang and co-workers also showed a similar trend. It was shown a pro-survival activity in colorectal CSCs relied on oxaliplatin-induced autophagy that participates in the maintenance of stemness and chemo-resistance [112]. A study also implicated that pharmacological blocking of factor Notch leads to autophagy activity and subsequent drug resistance in glioma neurospheres [113]. Taken together, more studies are required for establishing the impacts of autophagy function as a potential therapeutic target of CSCs.

## The correlation between autophagy and angiogenesis

Angiogenesis is a crucial phenomenon for maintaining blood nourishment under the normal condition that also participates in the regeneration of ischemic cardiac tissue or impaired peripheral vascular [114, 115]. In contrast, the tumor progression and metastasis could be enhanced by promoting angiogenesis under pathological conditions [116]. This phenomenon could participate in the healing of different organs and respond to the demands of tissues and organs under pathological and physiological conditions [117]. It seems that the angiogenesis potential of SCs has a critical role to accelerate restoration of injured tissues which is governed via cell differentiation into endothelial lineage and releasing different pro-angiogenic factors via paracrine manner [118]. It has been elucidated that angiogenic activity of SCs could be controlled by different intracellular mechanism peculiarly autophagy [119]. As a matter of fact, conduction of experiments to find the close relation between autophagy and angiogenesis seems to be critical in the context of tissue engineering and regenerative medicine.

### Direct effect of autophagy on ECs

Vascular ECs have a fundamental role in preserving normal functions of the cardiovascular organization [120]. Therefore, endothelial dysfunction has been distinguished as a corporate risk factor for almost all kinds of cardiovascular diseases. There is a growing body of literature attempting to understand the effect of autophagy on vascular pathophysiology [121, 122]. It has been well-documented that deficiency of autophagy may be a major mechanism and risk factor that elicits endothelial dysfunction and involved in the regulation of NO bioavailability [123]. In addition to a close relation of NO-autophagy, NO has a critical role in the regulation of mitophagy [124]. Studies demonstrated that autophagy-deficient ECs with blunt NO level promoting ROS and inflammatory cytokine production [125]. Along with the importance of endothelial maturation, endothelial and/or non-endothelial secretome have a pivotal role in the favor of angiogenesis [126]. The paracrine activity of endothelial lineage is mostly dependent on the secretion of factors by releasing exosomes [127]. The induction of autophagy was shown to increase the biogenesis of exosomes carrying endothelial specific factors such as vWF and VEGFR-2 [128, 129].

Emerging evidence also suggests that endothelial autophagy might modulate the uncoupling of eNOS. CAV1/caveolin-1 that is a critical factor of eNOS coupling and NO bioactivity is a key regulator of ECs autophagy [130]. Autophagy flux also embraces angiotensin-II utility required for endothelial function. It is well established that the detrimental effects of angiotensin-II on endothelial dysfunction may be limited by the autophagic system [131]. In patients with diabetes mellitus, one of the risk factors for cardiovascular diseases is vasculitis that occurs through several mechanisms such as hyperglycemia, amplified angiotensin-II production, and an augmented oxidative stress [132, 133]. Therefore, for applying to the clinical approach, it does necessitate establishing elementary in vitro studies for proving a protective effect of autophagy in restraining glucose-caused endothelial damages [133]. Recent investigations have demonstrated that some of the micronutrients with cardiovascular protective potential by enhancing autophagy activity in ECs. For instance, epigallocatechin gallate, resveratrol, and vitamin D were shown to trigger autophagy and diminish palmitic acid-induced gathering of lipid droplets, presumably via an increased lipophagy, lipid catabolism by the autophagosome-lysosome system [134]. Resveratrol, the main cardio-protective component in red wine, attenuates inflammation of EC via the promotion of autophagy [135]. A study showed that curcumin has the potential to induce autophagy and exert cytoprotective effects on EC survival in response to oxidative stress [136]. Also, vitamin D elicits cytoprotective effects in the endothelium by amplifying autophagic flux [137]. These data added a notion that the initiation of autophagy response is a compensatory defensive reaction following the onset of vasculitis.

Autophagy, besides of an indispensable role in the intracellular recycling of proteins and organelles, is also involved in protein secretion in distinct cell types such as beta cells, ECs, mast cells, intestinal paneth cells, and osteoclasts [129, 138–141]. Recent evidence suggests that autophagic vacuoles can be directly secreted by ECs under certain conditions such as starvation. Proteomic examination of these autophagic vacuoles revealed that they contain the high levels of vWF [142]. Exocytosis is one of the first lines of defense after vascular injury in ECs. Specific secretory vesicles within the ECs known as Weibel-Palade bodies contain numerous biologically active molecules, notably vWF [143]. It has been well-documented that Weibel-Palade bodies are frequently found near or within autophagosomes and endothelial autophagosomes. Pharmacological inhibitors of autophagy or knockdown of the essential autophagy genes, namely Atg5 or Atg7, inhibit the in vitro secretion of vWF, resulting in prolonged bleeding times. Autophagy, as a consequence, regulates endothelial vWF secretion, and transient pharmacological inhibition of autophagic flux may be a valuable strategy to prevent thrombotic events [129]. Literature point autophagy is insufficient in ECs isolated from individuals with diabetes mellitus. Moreover, Jessica et al. demonstrated that intact autophagy is essential for eNOS signaling in ECs. LA Rocca et al. declared that NO-mediated vasodilation was promoted by the induction of autophagy and highlighted that an impaired autophagy exacerbated endothelial dysfunction in old cells [144].

Under the diabetic condition, an impaired EC function could be reversed by the induction of autophagy [22]. Autophagy improved the angiogenesis potential of human umbilical ECs exposed to high glucose content by induction of tubulogenesis and cell survival activity [145].

Autophagy can be regulated in ECs by compounds circulating in the bloodstream or localized within the sub-endothelial layer of atherosclerotic plaque. In cultured HUVECs, for instance, vitamin D increases the level of factor beclin-1 [137]. Oxidized low-density lipoprotein and AGEs support the formation of autophagosomes [146, 147]. Endostatin, a powerful inhibitor of neovascularization in tumor treatment, initiates autophagic cell death in the human EC line [148]. Interestingly, GRP, an inducer of tubule formation, decreases expression of ATG5, Beclin-1, and LC3 [149]. These findings indicate that autophagy is required for the development of vascular ECs; its appropriate regulation is pivotal during fundamental adaptive responses such as secretion, cell proliferation, and other endothelium functions. Vion et al. previously demonstrated that inefficient autophagy contributed to the development of atherosclerotic plaques in low-shear stress areas, promoting inflammation, apoptosis, and senescent phenotype in ECs [150]. Because of the critical role of endothelial malfunction in the pathogenesis of the vascular-related disease, it would be told that the distractions of autophagy machine in ECs have noteworthy contributions [151].

**Fig. 2:**
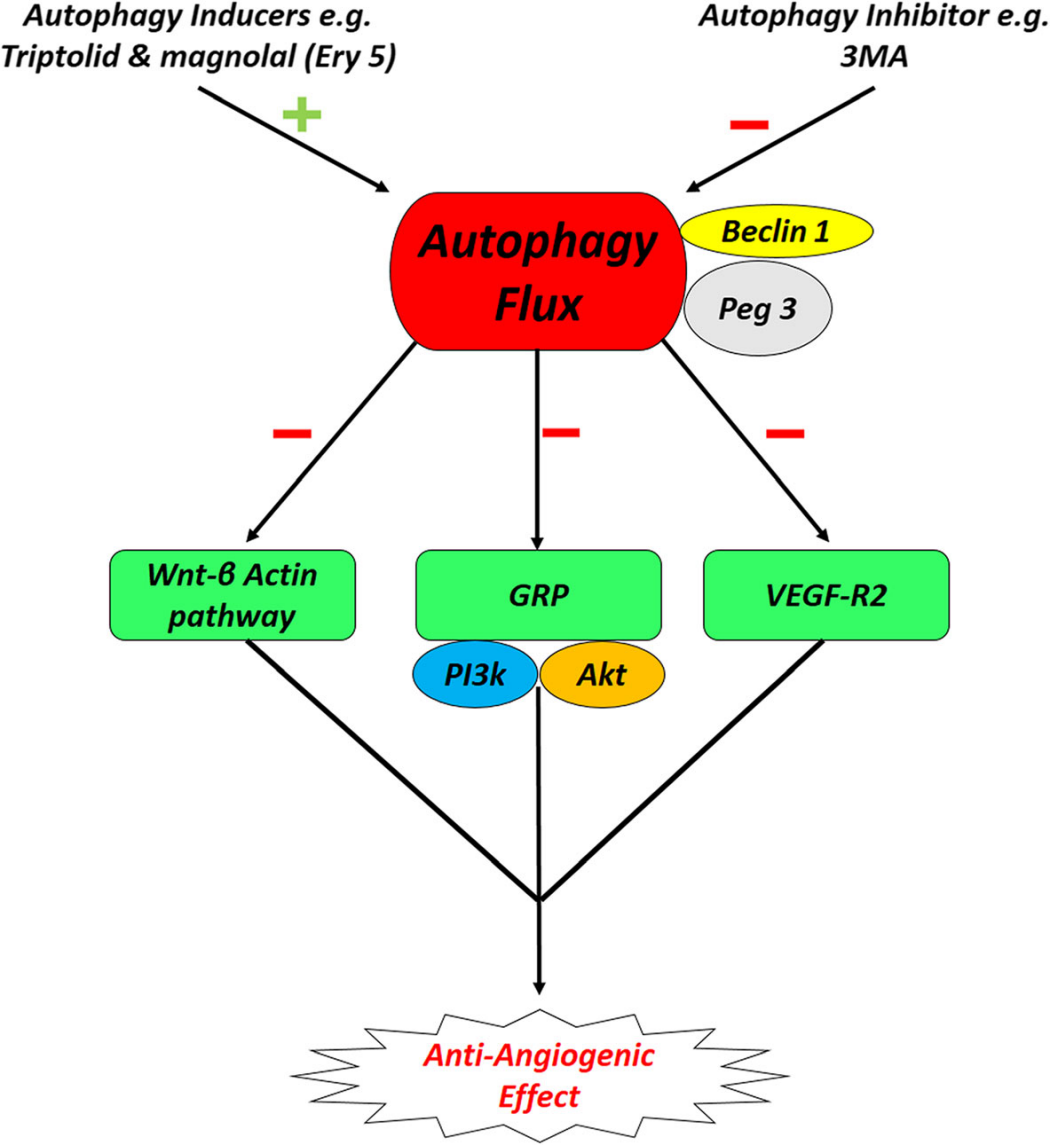
Representative image of the anti-angiogenic potential of autophagy. Anti-angiogenic effect autophagy is initiated via the modulation of Wnt/β-catenin axis. The application of autophagy inducer, magnolol (Ery5), has an inhibitory effect on migration and tube formation properties in both human umbilical vein endothelial cells and apoptotic resistant cancer cells while chemical blocking of autophagy by 3-MA or gene silencing of Atg7 and LC3 reversed anti-angiogenic effect of autophagy VEGFR-2 levels and angiogenesis are reduced following endothelial dysfunction and exposure to advanced glycation end products (AGEs) accumulation under diabetic condition. Autophagy plays a key role in VEGFR-2 degradation and impaired angiogenesis. Gastrin-releasing peptide (GRP) and its receptor GRPR through PI3K-AKT pathway have a pivotal role in cancer-related angiogenesis. By antagonizing GRPR, pro-autophagic proteins are overexpressed and angiogenesis blocked by autophagy-mediated GRP degradation mechanism

**Fig. 3:**
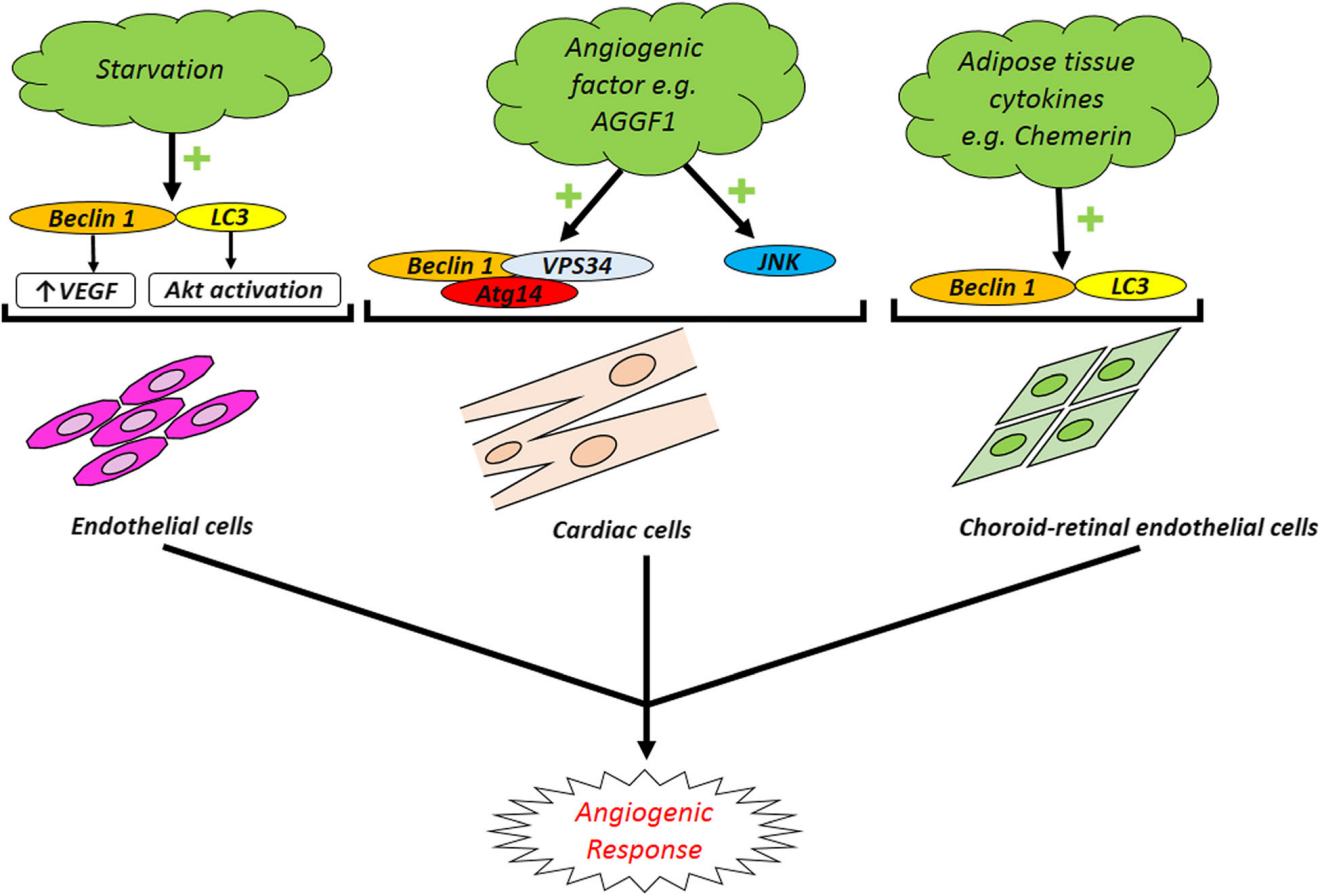
Angiogenic potential of autophagy. Starvation-induced autophagy triggers cell migration and in vitro angiogenesis by activating of VEGF and AKT protein on ECs. AGGF1-induced autophagy is a candidate for coronary artery disease and myocardial infarction treatment. Chemerin, a novel adipose tissue cytokine, also has a stimulatory effect on retinal endothelial cells angiogenesis by promoting autophagy activity and expression of the autophagy-related proteins LC3 and Beclin-1 increased during chemerin-induced migration and tubular formation

Based on the critical role of autophagy in the regulation of ECs’ function and angiogenesis, it seems that applying mechanisms which accelerate and/or inhibit autophagy signaling pathway could orient the pro-/anti-angiogenic behavior of endothelial lineage [151]. In recent decades, it was announced that autophagy stimulator/inhibitor could be applied as pharmacologically agents to modulate angiogenesis. Rapamycin, a mTOR inhibitor, shows anti-aging effects in the vascular system and promotes functionality of the endothelial system by increasing the remodeling rate in animal models [152, 153]. The prophylactic autophagy modulation seems to guarantee brain ECs against the apoptosis subjected to ischemic/reperfusion changes [154]. In this regard, some clinical trials were conducted in the manipulation of autophagy effectors to reach beneficial effects during the occurrence of cardiovascular disease. For example, the use of sirolimus was used as autophagy modulators after cardiac allograft transplantation (ClinicalTrials.gov identifier NCT01889992). Outstanding results lead to the prominent importance of autophagic flux for therapeutic approaches. By prescribing a vasodilator agent namely Icariin in ischemic disease, some evidence discovered the simultaneous correlation of endothelial secretion of vWF and induction of autophagy to promote angiogenesis [155]. It seems that the modulation of autophagy must be considered related to stage and intensity of injury. Some documents revealed that the inhibition of autophagy not only increased the angiogenesis rate but also suppressed the pro-angiogenic signaling pathways [156]. Zheng and colleagues reported that activation of Akt/eNOS axis inhibited autophagy and improved angiogenesis and cerebral ischemia-reperfusion injury [156]. A close association exists between autophagy machinery and the inhibition of angiogenesis response in various cells. The autophagic response outcome on tumor progression has been demonstrated in different cancer cell types such as osteosarcoma, glioblastoma, and colorectal cancers [157–159]. Wnt/β-Catenin pathway is a key regulator of cell proliferation, differentiation, angiogenesis, and cell death [160] (Fig. 2). However, autophagy induction presents an anti-angiogenic effect via modulation of Wnt/β-catenin signal pathway [161]. Additionally, the application of potent natural autophagy inducer, magnolol (Ery5), disclosed that excessive autophagy has an inhibitory effect on migration and tube formation properties in both human umbilical vein endothelial cells and apoptotic resistant cancer cells while chemical blocking of autophagy by 3-MA or gene silencing of *Atg7* and *LC3* reversed anti-angiogenic effect of autophagy [162]. VEGFR-2 levels and angiogenesis are reduced following endothelial dysfunction and exposure to AGEs accumulation under diabetic condition (Fig. 2). It was implicated that autophagy plays a key role in VEGFR-2 degradation and impaired angiogenesis [163]. Notably, it was shown that an excessive activity of autophagy is responsible for abrogated angiogenesis potential in MSCs exposed to diabetic serums by P62 overactivity [21]. Rezabakhsh and co-workers examined the pro-/anti-angiogenic potential of diabetic sera on human MSCs. They found that these cells lost endothelial differentiation in in vivo and in vitro conditions [21]. Likewise, the data declared an important correlation between overexpression of autophagic p62 aggregation and angiogenic factors in MSCs subjected to diabetic serums [21]. *Peg*3 one of the important genes encoding a zinc finger transcription factor contributes to tumor suppression. Upregulation of *peg3* increased Beclin-1 protein level and concurrently autophagy induction in ECs which inhibited migratory behavior and in vitro angiogenesis. On the other hand, *peg3* stimulates the secretion of thrombospondin-1, as a potent angiogenic factor, and modulates the angiogenesis independent of Beclin-1 transcription [164]. In neuroblastoma with highly florid vascularization, tumor progression and metastasis occurred rapidly with an achieved poor prognosis. Notably, GRP and its receptor GRPR through PI3K-AKT pathway have a pivotal role in cancer-related angiogenesis. By antagonizing GRPR, pro-autophagic proteins are overexpressed and angiogenesis blocked by autophagy-mediated GRP degradation mechanism [165] (Fig. 2). Autophagy is an essential survival pathway by stimulating angiogenesis during the imbalance of oxygen or nutrients supply achieved following anti-angiogenic pharmacotherapy of solid tumors. In this regard, it has been shown that hypoxia-mediated autophagy is considered as a novel mechanism of resistance to anti-angiogenic drugs during the cancer therapy [157]. In fact, angiogenic development of cancer cells under hypoxic condition leads to autophagy induction as a compensatory response via hypoxia-inducible factor-1a/AMPK pathway which increases cancer cell survival rates and drug resistance [159]. Interestingly, chloroquine as a pharmacological autophagy inhibitor could synergize the sunitinib anti-cancer effect by limiting angiogenic capacity probably through upregulation of inducible nitric oxide synthase, increased reactive nitrogen species production, and an increase of anti-oxidant capacity [166]. Moreover, starvation-induced autophagy in cultured BAECs triggered the cell migration and in vitro angiogenesis by activating of VEGF and AKT protein while the use of Atg-5 siRNA reversed the autophagy pro-angiogenic effects on BAECs [167]. It is noteworthy that retinal ECs have a contradictory behavior in comparison with ECs of peripheral vessels. In RF/6A cell line, autophagy as a positive regulator contributed to the hypoxia or high glucose-induced neovascularization under different stress conditions [167, 168] (Fig. 3). It has been found that chemerin, as novel adipose tissue cytokine, also has a stimulatory effect on RF/6A cells angiogenesis by promoting autophagy activity and expression of the autophagy-related proteins LC3 and Beclin-1 increased during chemerin-induced migration and tubular formation [169] (Fig. 3). Endostatin is one of the endogenous angiogenic inhibitors that limited the cell proliferation and migration. Both native and mutant (P125A-endostatin) form of endostatin could activate the autophagy machinery. During primary ECs treatment with P125A-endostatin, the levels of Beclin-1 increased while the apoptotic markers Bcl-2, Bcl-xL, and β-catenin decreased. By enhancing the β-catenin level and Wnt-mediated signaling, endostatin-induced autophagy was inhibited in ECs. Thus, autophagy is a survival response toward the endostatin-mediated apoptotic cell death [170]. Autophagy also was considered a potential angiogenesis-based therapy for cardiovascular diseases. AGGF1 is one of the essential angiogenic factor**-**induced autophagy through JNK activation and Beclin1-Vps34-Atg14 complex while the AGGF1-mediated migratory behavior, tubular formation, and aortic ring-based angiogenesis required autophagy activity. According to recent findings, AGGF1-based therapy is a candidate for coronary artery disease and myocardial infarction treatment [171] (Fig. 3).

### Role of autophagy on endothelial differentiation of SCs

To our knowledge, there are a few bodies of experiments targeting the potency of autophagy in controlling of SCs angiogenic orientation toward endothelial phenotype. The simultaneous activation of autophagy effectors and endothelial specific markers such as FLK-1, Tie-2, Cadherin-12, and Cadherin-19 was reported in human bone marrow monocytes after the induction of monocyte chemotactic protein-1. This finding highlights that autophagy actively participates and/or simultaneously is provoked during cell-to-cell trans-differentiation [172]. Li and co-workers showed that the promotion of autophagy via LncRNA WTAPP1 against miRNA 3 and 120 accelerated EPC maturation by modulating Akt/PI3K axis and type 1 metalloproteinase. These data highlight the critical role of autophagy on angiogenesis in either in mature or progenitor cell types [173, 174]. The beneficial effects of autophagy could direct the angiogenic potential of EPCs under diabetic condition by affecting migration and differentiation rate [175–177]. These findings propose that inadequate autophagy is a complicity mechanism for endothelial malfunction in diabetes mellitus [22]. Given the importance of endothelial dysfunction in the pathogenesis of atherosclerotic cardiovascular disease, these findings are probably to be clinically correlated [178]. Therefore, pharmacologic interventions directed toward recovering normal autophagic flux may have targeted therapeutic potential in related diseases. In another experiment conducted by Wang and co-workers, they documented that monotropein-inhibited autophagy through AMPK/mTOR signaling in EPCs led to an increased angiogenic differentiation and accelerated wound healing rate [179]. Considering the dual role of autophagy in cytoprotection and cytotoxicity of vascular cells, distinct targeting with careful titration will be required.

### Role of autophagy on angiocrine activity of SCs

In addition to trans-differentiation behavior, SC paracrine activity is pivotal in the induction of angiogenesis in the target tissues [180]. MSCs treated with autophagy stimulator such as rapamycin potentially secreted a high level of VEGF via the modulation of ERK phosphorylation and thereby accelerated the regeneration of wound healing. The ablation of autophagy signaling by using si-Beclin-1 blunted pro-angiogenic effects [181]. In a study, preconditioning of rat MSCs with low-energy shock wave therapy promoted the autophagic status with activation of PI3K/AKT/mTOR and NO/cGMP pathways also increased the VEGF factor after transplantation to erectile dysfunction model [119]. The co-culture of periodontal ligament MSCs with ECs exposed to inflammatory condition showed a better angiogenic behavior. High levels of MSC-derived bFGF and angiogenin factors were detected [182]. In line with these reports, autophagy accelerates angiogenesis-promoting capacity of SCs even in the inflammatory environment. It should be noted that autophagy activation not only promotes the angiocrine capacity of SCs but also activated abnormal conditions. For example, it was shown that EPCs autophagy response is activated after being treated with high glucose content (30 mM) or hypoxic condition [183, 184]. The activation of autophagy decreases the number of apoptotic cells and forces cells to tolerate insulting conditions. Commensurate with these comments, autophagy is able to increase the paracrine activity of SCs in response to various conditions.

## Conclusion

In conclusion, findings strongly clarify that autophagy dual effect on angiogenic behavior is entirely dependent on cell type, cellular demands, and other conditions which need to be evaluated by further clinical trials and more comprehensive tests.

## Abbreviations

3-MA: 3-Methyladenine
AGEs: Advanced glycation end products
AMPK: Adenosine monophosphate kinase
ASCs: Adult stem cells
ATG: Autophagy-related genes
BAECs: Bovine aortic endothelial cells
CMA: Chaperone-mediated autophagy
CPCs: Cardiac stem cells
CSCs: Cancer stem cells
ECs: Endothelial cells
EPCs: Endothelial progenitor cells
ESCs: Embryonic stem cells
FGF: Fibroblast growth factor
GRP: Gastrin-releasing peptide
HMBOX1: Homeobox-containing 1
HSCs: Hematopoietic stem cells
LAMP2A: Lysosomal-associated membrane protein type 2A
LIF: Leukemia inhibitory factor
MPCs: Muscle progenitor cells
MSCs: Mesenchymal stem cells
mTOR: Mammalian Target of Rapamycin
NO: Nitric oxide
NSCs: Neuronal stem cells
PI3K: Phosphatidylinositol 3-kinase
PTEN: Phosphatase and tensin homolog
RF/6A: Rhesus macaque choroid-retinal endothelial
ROS: Reactive oxygen species
SCs: Stem cells
SIRT-1: Sirt1
SNARE: Soluble *N*-ethylmaleimide-sensitive factor attachment protein receptor
ULK1: Ubiquitin-like kinase 1
VEGFR-2: Vascular endothelial growth factor receptor 2
Vps: Vesicle-mediated vacuolar protein sorting
vWF: Von Willebrand factor
